# Rabies1A clinical lecture delivered at the Veterinary Department of the University of Pennsylvania.

**Published:** 1889-01

**Authors:** Rush Shippen Huidekoper

**Affiliations:** Vet.; Professor of Veterinary Anatomy and Pathology


### Art. V.—RABIES.[1]

BY RUSH SHIPPEN HUIDEKOPER, M. D., VET.

*Professor of Veterinary Anatomy and Pathology.*

Gentlemen :—I take this opportunity of making some remarks upon hydrophobia in view of the fact that the disease has existed in an almost epizootic form during the past two months, in view of the importance of the subject, and of the absolutely false ideas which prevail in the popular mind in regard to hydrophobia, which has led to the definite statements and the expressed belief on the part of reputable physicians, that no such disease exists, simply because they have not seen cases, and because the cases are so excessively varied in their symptoms and their course. The disease is subtle and the symptoms of derangement of the highly organized nervous system may be different in different cases. In one case there may be excitability, in another depression, while in others there may be alternating conditions of anæsthesia and hyperæsthesia involving both the central nervous system, the two sets of peripheral nerves, the motor and sensory and the sympa-

[1] A clinical lecture delivered at the Veterinary Department of the University of Pennsylvania.

thetic system. In view of these considerations, I thought it advisable to take up the consideration of this disease out of the regular course where it belongs, that is with the contagious diseases.

I shall in the beginning adopt one of the synonyms in place of the more common term of hydrophobia. I prefer the name rabies, meaning to be mad. We have as synonyms, *hydrophobia*, the fear of water, again madness, in the Greek, *lyssa* ; in the German, *hundswuth* or *tollwuth*, in Italian *rabbia*, in French, *la rage*, in Spanish, *rabia*. In addition we have in all languages the term hydrophobia, the fear of water.

*Rabies* is a virulent and contagious disease especially of the canines, felines and vulpines, but inoculable in other animals both wild and domestic. The disease has been propagated to wolves, it has been seen in skunks, in raccoons and in badgers. The disease while most common in the species enumerated, is communicable by inocculation to all other species of animals including man and the birds. Birds after inocculation suffer from severe nervous symptoms, but rarely die. The disease is almost invariably fatal in other animals.

Rabies is a disease attended with marked derangement of the nervous system both central and peripheral. The derangement may manifest itself by increased excitability, by diminished excitability and paralysis or with perversion of the nerves and special senses.

This disease is referred to four centuries before the Christian era by Democritus. It is also referred to by Aristotle and Plutarch. The first distinct description of the disease was that of Celsus fifty years before the Christian era. He refers to it under the name of hydrophobia and recommends the preventive treatment which is the only one that we can adopt, that is excision, cauterization and suction of the wound with the mouth in order to remove the poison. Again hydrophobia or rabies is referred to by Discordides, Pliny and Galen. The description of it given by Galen is rather a lengthy one. Since the ninth century, it has been believed that the disease could be relieved by St. Hubert, the patron-saint of hunting dogs, and pilgrimages to the shrine of St. Hubert were believed to be beneficial in the case of those bitten by rabid dogs. The disease was known by the Arabs in the twelfth century, and in one of the earliest books on horses "El Raceri," we find the description of a disease communicable by bites from dogs to horses. In the seventeenth century the disease was studied by Hamel and Boerhaave, and later by the great surgeon Hunter who was the leader in the studies of inflammation. In the last century the disease was studied by Majendie, Hertwig, Youatt and others. During the past few years rabies has been made the subject of special study. The most extensive works that have been written upon this subject are those of Bouley in French, and those of Fleming in English. These are the principal authorities to which you can refer. It has also been studied by Leblanc and in the last few years its mode of development and its prophylaxis has been especially studied by Pasteur.

At the end of the sixteenth century this disease assumed an an epizootic form throughout the whole northern part of Italy and the countries bordering on the Adriatic, extending down the Danube into Hungary and Turkey. It reappeared in Hungary in 1714, that is about a century later. It appeared in epizootic form in connection with anthrax in central Europe in 1691 and 1722, so much so that it was supposed by some that there might be some connection between the two diseases. In 1590, 1604, 1719, 1759, 1763 and 1774, it occurred in epizootic form throughout parts of Europe and in England. The medical writers of this time pay considerable attention to the disease. At the end of the eighteenth century it appeared in North America and the West Indies. In 1803 and 1834 it occurred throughout Switzerland in an epizootic form attacking a large number of animals. For the last century, the disease has been increasing in western Europe. It is common in Spain although excessively rare in Portugal. It is comparatively common in England. It is much rarer in Scotland and in Ireland. The disease is known to occur in the Arctic regions. Both Kane and Hayes, the Arctic explorers, give us descriptions of outbreaks of rabies in Southern Greenland among the dogs of the Esquimaux and also among wild animals. So great have these outbreaks been that the Esquimaux have been driven from certain sections, being dependent upon their dogs as a means of travel. During the present century there have been several epizootics among the wild wolves of Russia and a large number of people have had the disease as a result of being bitten by these animals. In 1838 there was an extensive outbreak in Denmark, and later in 1842, in Vienna. In 1860, there was a severe outbreak in Lancashire, which is in the neighborhood of Liverpool. So great was the outbreak that one thousand dogs were destroyed by the authorities in order to prevent the spread of the disease. In 1872 there was another extensive outbreak which was studied by Fleming. The severity of outbreaks of this disease varies. One or two animals suffering with the disease biting other animals serve to spread the contagion, and as in other contagious diseases, the epizootic spreads until some accident stops its course or the number of animals to be inocculated diminishes.

Rabies is unknown in Australia, in Van Dieman's Land, in the Azores and in St. Helena. In other islands as in Iceland it occurs frequently. In those islands however where the disease has never been imported, it is unknown. It was brought to America and to the West Indies only at the close of the last century.

Zundel says of this disease that it is scarcely possible to find two cases which present exactly the same signs and symptoms. It is a disease which is greatly influenced by the race, temperament, sex, age and the individuality of the animal affected. We find in different races of dogs, that the disease will greatly differ. The affectionate, intelligent dogs will do less damage and are more apt to have the disease in a less ferocious form than dogs of a ruder sort.

The older authors divided rabies into some six or seven varieties. At present we divide the disease into three forms : furious, mute or paralytic, and that known as the mixed form. The latter is the most common. In this we have symptoms of the furious form ending in those of the mute or paralytic form.

The etiology we shall to-day leave to one side as we have not time to take it up, except to say that we distinctly assume that rabies comes from one cause alone and that is contagion. We apply here the same rule as in all other contagious dieases, where there is doubt, to asume that contagion is the *only* source of origin as the safest thing for the animal and the safest means of checking the spread of the disease by sanitary legal measures. We shall therefore assume that the disease comes only from contagion. The possibility of the spontaneous development of rabies has been thoroughly studied, but no evidence has been found to support this view, and in the immense majority of cases the origin can be distinctly traced to direct contagion.

After the inoculation there is a period of incubation varing from seven days to seven and one-half months. I find that Fleming gives several cases which he claims to be authentic where the period of incubation has been over one year. In one case which I saw myself, the dog was kept in absolute confinement for seven and one-half months after being bitten, where it was impossible for it to receive contagion. He was considered so safe that the owner had been requested to remove him from the hospital, but he developed the first symptoms of the disease on the day that he was to be removed. The ordinary period of incubation is about three or four weeks. Remember, however, that it may be as short as seven days or as long as seven months, and if this long, it may be a year or more.

The symptoms of rabies are generally those of nervous irritation with agitation of a part of the body as of the head or of the extremities. The animal is restless and a little excitable. The disease may, however, appear with symptoms of nervous depression. The animal may be quieter than usual and become caressing to its owner, so much so that a dog becoming unusually affectionate for a period of several hours should be looked upon with suspicion. At the outset there is a tendency for the dog to lick any cold object, as the floor, a piece of china or a stone. We find the animal becoming dejected, rather more sober and quiet in its habits, simply as if not well. This is so in any form of disease, but in addition we see a constant restlessness which does not occur at the beginning of other diseases. When a dog is depressed from pneumonia or pleurisy and becomes quiet he remains so. A dog who has become depressed at the commencement of rabies will become quiet, then throw up his head as if snapping at a fly and then become quiet again. He gets up without apparent cause and again lies down. After getting curled up, he gets on his feet again. Exceedingly depressed, in the intervals, he shows alternate periods of agitation. These may be so slight as to be scarcely noticeable unless looked for carefully. You have seen these symptoms in the cases which we have recently had, and they were most marked in the last case, that of the setter.

At the outset, we also find a tendency for the animal to scratch and tear things without any reason. That was the history in the case of the spaniel. This dog after being excessively quiet suddenly jumped on the bed pulled off the pillow and tore up the bed clothing, then went into a corner and quietly laid down. We find the animal sniffing at natural articles with which he is familiar, as the carpet, chairs, and other articles of furniture. He sniffs as though taken to a strange place or where there has been another dog. We find that the animal minds his master when spoken to throughout the disease, but he seems to forget and is slow in his movements. He will obey, but in a moment seems to forget what he was told. One of the two cases which we had here two years ago was a black and tan terrier. He would mind his owner as long as he was watched, but the instant the owner would turn to speak to some one, the dog would sneak behind him and try to bite his legs. As soon as the owner would look at him, he would again come to the front. He would have bitten me if I had attempted to handle him and he would have bitten his owner if he had not been watched.

The animal with rabies has delusions, especially of the special senses. The optic nerve is irritated so that the animal sees lights and objects that do not exist. The eighth pair is also irritated so that the animal hears imaginary sounds and natural sounds are exaggerated. All kinds of sounds and strange objects will increase the irritation in dogs suffering from rabies. Sometimes there seems to be intense pain in the ear, and again in the region of the throat, or in both situations. The animal will be found scratching at its ear as if it had auricular catarrh but on examination of the ear, nothing abnormal is found. The animal will scratch at its throat as though a foreign body was lodged there. This is one of the most common symptoms at the outset. The animals which you have seen, you will remember were brought here with the history of having a bone in the throat.

There are absolutely no hydrophobic symptoms. Fleming says that there is no dread of water in the rabid dog. There is from the outset of the disease no fear of water. The rabid animal is thirsty and would drink it it could, and will attempt to drink. We saw this in two of the cases confined in the iron cage here. Both animals dipped their tongues into water, but left it as they were unable to drink on account of the paralysis of the throat. At the commencement of the disease, the dog usually drinks a good deal, but later it is unable to drink. At the outset, the animal takes his ordinary food, but at a later period he leaves ordinary food to take foreign articles by preference. We find it eating straws and sticks. In the last case the dog eat up its muzzle and leather collar. They will eat stones, the manure of a horse and their own faeces, and will drink their own urine. They will eat dirt from the street and dig up sods and eat the dirt from the roots.

At the outset there is vomiting and frequently vomiting of blood. This bloody vomiting is not due simply to the presence of foreign bodies in the stomach, for it has been seen among earlier symptoms in man where no foreign bodies have been swallowed. It has, however, been seen only at the outset of the disease.

In the early period of the disease there is profuse saliv ti on. At a later period the saliva is frequently scanty so that the mouth is dry. The popular idea is that in later periods of the disease, there is profuse salivation, that the mouth and lips will be frothy and that the saliva will drip from it. You will remember that in the cases bronght here the mouth was perfectly dry, the tongue hung from the mouth, and the lips and tongue were covered with dirt and not moist. Remember that the salvia may flow in excess or it may be absent.

There is a change in the voice of the dog, which has been called by Bouley, the *voix de coq*. This is a peculiar hoarse sound which resembles the crow of the cock. The bark resembles to a certain extent the howl given by a hound lost from the pack. It commences with a low base sound and ends in a sharp high sound. Another peculiarity of the bark is that it is repeated from five to eight times in rapid succession. The animal is then quiet for a little while, and then the bark is repeated. The howl of the lost dog is never repeated more than two or three times in succession, and the change from the low to the high sound takes place much more slowly than in the dog with rabies.

Among the early signs of rabies is the presence of *lyssa* or the vesicles of Marochetti. These are present in only about fifty per cent. of all cases and are small vesicles, perhaps little lymphatic tumors seen on the surface of the mucous membrane of the mouth. As they are so frequently absent, they are not of much service in diagnosis.

The sensation of the animal is diminished. The hydrophobic animal does not feel pain as the sound animal does. We see this in twisting the ear in order to hear the voice. In the case of the setter which you saw the ears could be twisted for some minutes before the animal would show signs of pain. Ordinarily the animal would have howled at the first touch. I have seen a hydrophobic animal take hold of a red hot bar of iron and hold it as he would a stick, without evincing any pain. In this case the animal was in a furious state.

There is frequently in this disease a pruritus, that is a hyperanæsthesia in the neighborhood of the pharynx that causes the scratching at the throat. The animal will scratch at the old scar until it breaks out in an open sore. In the horse this is almost invariably present. It is not so common in the dog. In the hog again, it is quite frequent. We also find irritation of the genital organs attended with priapism and emission of seminal fluid. This is found in all animals, but it is more common in the case of cattle than in other animals.

In the second stage of rabies we may have the animal developing a greater excitability and becoming furious. The eyes will have a staring look as if fixed intently upon some object, but upon no definite one. This was well seen in the setter. That animal traveled for hours around his cage until in fact all his bedding disappeared. If you came into the kennel, or spoke to him attracting his notice, he would look at you intently, and then his eyes would wander off with the same look, but directed toward no definite object. This is one of the very characteristic symptoms. The pupils are invariably dilated. The animal seems forgetful. He will attack another dog, and after biting him once or twice, bears no malice against him, but is just as willing to turn around and bite another dog or some other animal. There is apparently no memory during the excitable stage. There will however be intervals of intelligence. After wandering for a period, he will remain quiet for a time, but will start up if disturbed, especially by any sudden sight or sound. In the furious stage the dog prefers to bite another dog rather than any other animal. A dog with a man acts as a safe-guard against a rabid dog. The mad dog will bite the dog in preference to biting the man.

In this excitable state the dog travels until exhausted or until commencing paralysis forces it to again become quiet, when, if it has the strength, it will frequently return to its home.

The second stage may be one of paralysis instead of a furious condition. After premonitory symptoms which are excessively difficult, the animal quietly curls in a corner, is affected with paralysis and dies without ever having been furious, or as in one case which we had, the animal would have been furious if it had had the strength. It would snap at things, but on account of the paralysis of the jaw, it had no strength to hold on. In the mute or paralytic form we have the throat trouble most marked, and this is followed at once by paralysis of the lower jaw and of the tongue. Most of the cases which we have recently seen have been of this form. In this form, in the absence of saliva, the tongue becomes dry and covered with dirt. The animals are restless, turn round and scrape up their bedding, and present the peculiar staring fixed look which is seen in the furious form. Yet the animal will want to be caressed by the owner. There may however be salivation at the outset. The appetite is preserved, but the animal is unable to swallow. It will go to its food and pan of water and attempt to eat and drink, but is unable to do either. The pharynx is choked with all sorts of foreign matter. Inside the pillars of the throat and between the back teeth and the cheeks, will be found foreign matter which the animal has attempted to swallow. After a time the paralysis becomes complete and the jaw drops open, remaining in that position.

The sight of other dogs does not excite a dog with mute hydrophobia as it does a dog with the furious form of the disease. In the former variety there is little or no tendency to bite. If there is a tendency to bite, the dog is unable to exert any amount of force on account of the paralysis of the muscles and, hence its bite is innocuous. In the mute form the paralysis of the throat extends to other portions of the body until the animal is totally paralysed and dies in this condition.

In other animals the disease occurs with rather more distinctive symptoms and without the great variability that is seen in the dog. In the horse, the disease can almost be traced to inoculation. One of the first symptoms in the horse will be found to be irritability at the point of inoculation. If, for instance, the bite has been on the lip, we will find that the first sign of the development will be that the horse rubs the perfectly healed scar against the manger, and in the course of half an hour there will be a large sore where the bite occurred. The horse refuses food, becomes nervous, turns its head rapidly, watches the entrance of the stable attendant, and watches what the other animals in the stable are doing. From this the horse goes into a furious stage, biting at anything within reach, kicking at any other animal or man that approaches, and does this with perfect reason. When a rabid horse kicks it looks where it is kicking. When he attempts to bite he knows what he is trying to do. This enables us to make a perfectly clear differential diagnosis between rabies in the horse and the fury which attends meningitis. In the latter affection, the horse is just as furious, but it makes little difference to it what it kicks. A man entering the stall of a horse suffering with meningitis is perfectly safe if he keeps carefully to the side where he can avoid the kick or the bite. Two men can readily manage such a horse by guiding it from each side. The animal kicks and bites without knowing what he is doing. In the rabid horse there is also venereal excitement, priapism, and in the stallion emission of seminal fluid. The horse invariably dies in a short time from traumatism, as from fracture of the upper jaw, with hemorrhage, or falling and breaking a leg or the pelvis back, or producing hemorrhage in some other way. Throughout, the horse is in an excitable state, although this may end in paralysis.

In cattle we have the excitable condition. The animal commences to bellow, to paw and tear up the ground with its horns, tear at the fences, and in the stable at anything within reach of its horn. The eyes have the same staring expression that has been referred to in the case of the dog. If the animal does not die from traumatism, paralysis comes on in five or six days, during which there have been almost continuous successive attacks of fury with certain intermissions of tranquility.

In sheep there are the same general symptoms. The animal becomes excited butting with its head and with its horns if it has any, but neither in sheep nor in cattle do we have any tendency for the animal to bite. The horse bites and strikes with its forefeet, which in that animal serve as weapons of offense and defense. The other animals use only their ordinary arms, their horns and feet. Cases have been seen in which sheep would eat animal food and drink blood during the course of the disease, animal food being as unnatural for the sheep as foreign bodies for the dog.

In pigs there is intense pruritus at the point of inoculation. The animal shows general malaise, and a period of discomfort, is restless, and looks around. It finally becomes excessively savage toward any other form of animal. A sow with small pigs in the intervals between the attacks of fury, will be much more attentive to her young, and caress and suckle them more than when in a state of health. In the furious stage she will injure her young just as she would any other animal.

The lesions of hydrophobia are nearly all secondary. The *lyssa* or vesicles of Morochetti are not always present, as has already been stated. Of course where the dog has obtained foreign matter, it will be found in the stomach. In considering this subject I shall confine myself to the lesions found in the dog. The presence of foreign matter is not absolutely diagnostic, for we sometimes find the same condition in gastritis and enteritis. There is usually, and in the paralytic form always, intense congestion of the throat, palate, pharynx and larynx. There is general congestion and usually œdema of the muscles of the pharynx and larynx in the paralytic condition. We find also congestion of the internal organs, especially of the membranes. We find petechiæ in the walls of the stomach, in the intestine, in the mesentery. We find petechiæ and ecchymotic spots in the lungs. We find the brain in a congested condition, especially the isthmus that is from the corpus striatum back to the medulla. There is also intense congestion in the back part of the medulla, that is commencing with the roots of the fifth pair of nerves and extending to the hypoglossal. The nerves most markedly affected are the fifth, seventh and eighth pairs, and hypoglossal nerves. The sixth the least. There is little irritation on the part of the pneumogastric either as indicated by the symptoms or by the post-mortem results. The glosso-pharyngeal nerve is also involved.

Under the microscope there is found to be proliferation of the white cells, and of the amœboid cells in the white matter, but more in the gray matter in the posterior part of the medulla. These are the only lesions that we find that are of any importance. The lesions of hydrophobia were first thoroughly studied by Eckel and Brückmuller whose investigations were made in Vienna.

The poison or contagium of hydrophobia or rabies, which we assume to be the only cause of the disease is certainly propogated throughout the nervous system. Inoculation of the blood rarely gives any definite result. The virus is eliminated by the salivary glands, so that the saliva is capable of producing the disease in other animals. Again the poison is found in the nervous system, in the medulla, and in the anterior part of the spinal cord. As you saw recently the inoculation of a portion of the spinal cord from a rabid dog into the subcutaneous tissue of the neck of a sound dog produced the disease in nineteen days. That there exists in the medulla a poison capable of producing the disease, a poison which will be attenuated by exposure to the air, was shown by Pasteur. The poison in the saliva which is at first virulent disappears to a great extent in the course of twelve hours after death, that is by the time that the body has become cold. The poison remains in the nervous system so long as decomposition has not taken place, and the part is surrounded by carbonic acid and is protected from the air. It will remain active at least six weeks in an unopened dog buried in a cold grave. On exposure of the spinal cord to the air, the poison gradually becomes attenuated and finally disappears.

Pasteur took a piece of medulla from a rabid dog, cut it into sections and placed each piece in a separate jar numbered from one to fifteen. In each jar was placed a small piece of caustic potash to absorb any moisture which would favor decomposition. It is found that at the end of fifteen days exposure, the nervous mass has lost its virulence. At the end of fourteen days it still exists but in a very attenuated form. So, it gradually increases in virulence until the cord which has been kept only two or three days is as virulent as that directly from the animal. Pasteur found that when the inoculation was made directly from the animal the period of incubation was not the same, due probably to varying intensity of the poison in different cases. It was found that some died seven days after inoculation, some ten days, some fifteen, and some lived as long as three weeks. By successive inoculations from rabbit to rabbit it was found that the period of incubation was shortened, and when a series of twenty-two inoculations had been made, it was found that the period of incubation was fixed, and did not vary six hours from seven days, the extreme being six days and eighteen hours and seven days, and six hours. This fixed virus was then kept from one to fifteen days. It was found that from the first to the eighth day it would invariably produce the disease in a varying period of time, sometimes a week, sometimes two weeks, and at times even three weeks. It was found that the weaker virus, that kept from the eighth to the fifteenth day, would not produce the disease. It was found that if the weak virus, from the thirteenth to the fifteenth day were inoculated, and this followed by the inoculation of the stronger virus the disease would be longer in developing. It was also found that by following the inoculation of the material kept thirteen to fifteen days by that kept ten to twelve days, the disease would not appear. This was followed by the inoculation of material kept from three to eight days, and then by that kept one day, and this by virus direct from the animal, and yet rabies would not be produced, while inoculations of the same material into animals not previously inoculated would produce the disease. We have therefore ample proof of the presence of a virus capable of attenuation and self protection just as we have in the attenuation of the bacillus anthracis by lengthy exposure and heat. It was also found that these inoculations could be made with considerable rapidity, and could be made after the animal had been inoculated subcutaneously with the pure virus, and that a series ot these inoculations would serve as a protection. These experiments were carried to such a stage that Pasteur had in his labratory fifty dogs that were absolutely refractory to inoculations which would produce the disease in dogs not previously inoculated with weaker viruses.

At this stage of his investigations with the authority, and the advice of one of the most prominent physicians of Paris, Pasteur undertook these inoculations on the human being, which have been extended to a large number, but with rather doubtful results. The investigations of Pasteur have certainly aided us greatly in the recogniticn of the nature of the disease, but the success of his prophylactic treatment is certainly still a question.

A large number of persons who have submitted to these inoculations have died of hydrophobia, and with our present knowledge of the mode of propogation of the poison, it is certainly a question whether or not the disease has not been communicated by the inoculations which have been made for purposes of protection.

